# The All-Cause Mortality and a Screening Tool to Determine High-Risk Patients among Prevalent Type 2 Diabetes Mellitus Patients

**DOI:** 10.1155/2018/4638327

**Published:** 2018-07-11

**Authors:** Mohamad Adam Bujang, Pei Xuan Kuan, Xun Ting Tiong, Fatin Ellisya Saperi, Mastura Ismail, Feisul Idzwan Mustafa, Abd Muneer Abd Hamid

**Affiliations:** ^1^Clinical Research Centre, Sarawak General Hospital, Ministry of Health, Kuching, Malaysia; ^2^Health Clinic Seremban 2, Ministry of Health, Seremban, Malaysia; ^3^Non-Communicable Diseases Sector, Ministry of Health, Putrajaya, Malaysia; ^4^National Clinical Research Centre, Ministry of Health, Kuala Lumpur, Malaysia

## Abstract

**Aims:**

This study aims to determine the all-cause mortality and the associated risk factors for all-cause mortality among the prevalent type 2 diabetes mellitus (T2DM) patients within five years' period and to develop a screening tool to determine high-risk patients.

**Methods:**

This is a cohort study of T2DM patients in the national diabetes registry, Malaysia. Patients' particulars were derived from the database between 1st January 2009 and 31st December 2009. Their records were matched with the national death record at the end of year 2013 to determine the status after five years. The factors associated with mortality were investigated, and a prognostic model was developed based on logistic regression model.

**Results:**

There were 69,555 records analyzed. The mortality rate was 1.4 persons per 100 person-years. The major cause of death were diseases of the circulatory system (28.4%), infectious and parasitic diseases (19.7%), and respiratory system (16.0%). The risk factors of mortality within five years were age group (*p* < 0.001), body mass index category (*p* < 0.001), duration of diabetes (*p* < 0.001), retinopathy (*p* = 0.001), ischaemic heart disease (*p* < 0.001), cerebrovascular (*p* = 0.007), nephropathy (*p* = 0.001), and foot problem (*p* = 0.001). The sensitivity and specificity of the proposed model was fairly strong with 70.2% and 61.3%, respectively.

**Conclusions:**

The elderly and underweight T2DM patients with complications have higher risk for mortality within five years. The model has moderate accuracy; the prognostic model can be used as a screening tool to classify T2DM patients who are at higher risk for mortality within five years.

## 1. Introduction

The incidence and prevalence of type 2 diabetes mellitus (T2DM) especially among adults are increasing over time [1–3]. By the year 2030, it was estimated that the prevalence of T2DM patients was projected to be at 552 million globally [4]. Besides population growth, this increase of T2DM has been linked to aging population, urbanisation, obesity, and physical inactivity [5, 6]. Hence, T2DM is a noncommunicable disease that has become a pandemic. T2DM can lead to serious complications and mortality if the disease is not treated early and appropriately.

Diabetic patients experienced earlier mortality as compared to nondiabetic individuals of a similar age [7]. Therefore, it is necessary to identify and categorise T2DM patients who are at a higher risk for mortality. The ultimate aim in identifying this group is not only for early detection but also to provide appropriate attention and early interventions for these patients to reduce the mortality rate. Premature mortality can be prevented when appropriate treatments, healthy lifestyle modification, and proper counselling are provided to these patients.

Only few studies regarding predictive modelling for mortality among T2DM patients have been conducted but there are limited study on predictive modelling for mortality among T2DM patients in Southeast Asia region [8–11]. Majorities of previous prognostic models were developed based on cox regression model. Looking on the condition of data such as cohort and when the duration of diabetes is known, cox regression could be the best option for modelling when the duration of disease is treated as part of the outcome. However, if the duration of disease is treated as part of the contributing factors, logistic regression is appropriate [12–14].

This study aims to determine the all-cause mortality and the associated factors towards mortality within five years among patients with T2DM regardless of years diagnosed or duration of disease. Following that, a screening tool was developed to screen potential risk of mortality within five years' period among patients with prevalent T2DM. The screening tool can be used to classify patients who are at high risk of mortality whom will require more medical attention and also to raise the awareness among the public.

## 2. Methods

This is a cohort study using secondary data from a national diabetes registry (Malaysia). The demographic and clinical details of T2DM patients were captured from an Audit of Diabetes Control and Management (ADCM), Malaysia, between 1st January 2009 and 31st December 2009. This registry has obtained approval from the Medical Research Ethics Committee (MREC) and the full details of the study design, methods, and recruitment for the ADCM have been published elsewhere [15].

ADCM is a national diabetes mellitus registry with the aims to provide information about outcomes of diabetes care and to facilitate health care policy making in this area. The registry is an online registry database started in May 2007 which included all T2DM patients aged 18 years old and above from both government health clinics and government hospitals in Malaysia. The approval for using the data for this study was obtained from the committee of ADCM.

This clinic-based prospective cohort study recorded a total of 70,899 T2DM patients. Characteristics of these T2DM patients were with minimum age of 18 years and notified from government health clinics throughout Malaysia. The demographic profile, risk factors, and clinical parameters were recorded according to a standardised protocol [15]. Their records were matched with the national death record (NDR) at the end of year 2013 based on national Identity Card (IC) number (unique number assigned to each Malaysian citizen) to determine status of mortality within five years and also the causes of death based on ICD-10, 2016 [16].

### 2.1. Statistical Analysis

Out of 70,889 records, 69,555 records were analyzed after excluding duplication cases. Descriptive analysis was conducted to determine the cause of death based on ICD-10 codes. A univariate analysis such as Pearson chi-square test was applied to determine the significant parameters associated with the mortality within five years. Eighteen parameters were tested toward the status of mortality within 5 years. Considering the dataset was extremely large, the significant factors from the univariate analysis with *p* < 0.05 and having odds ratio more than 2.0 were selected for the parameters to be included in the multivariate analysis.

For the purpose of multivariate analysis, the dataset was first divided into three sets where the first set for model 1 consists of 60.0% of the overall dataset, the second set for model 2 consists of 40% of the overall dataset, and the third set for model 3 included all subjects. The sample selection for model 1 and model 2 were taken consecutively based on date of notification into the registry. The ideology behind such selection is to ensure the analysis and results on the later data (the latter 40% of the subjects) is consistent with the earlier data (the earlier 60% of the subjects), to reflect ongoing situation and timeline. Based on this sampling method, the consistency of the results will indicate that the magnitude of the contributing factors toward the outcome is about the same irrespective of time. Hence, sample selection based on consecutively sampling was chosen instead of random sampling.

Using the same set of parameters (determined from the univariate analysis), multivariate analysis was conducted based on logistic regression using forward likelihood ratio method. The cutoff probability for variable selection was set at 0.05 for both inclusion and exclusion criteria. The coefficients, odds ratio with respective confidence interval and *p* values, were recorded.

First and foremost, multivariate analysis was conducted using logistic regression for model 1 and model 2. After ensuring that there was no obvious difference in terms of the effect sizes based on results from model 1 and model 2, thus model 3 which included all subjects was analyzed and used as the proposed model for screening tool. The model 3 was selected because the aim to develop a screening tool should be based on large dataset since a large dataset will provide more information. Hence, the coefficients that were derived from the whole dataset are more stable and reflect to the targeted population.

A screening tool to screen high-risk patients (mortality within five years) was formulated using the logistic regression model. The equation model was derived based on the selected variables' coefficients based on the logistic regression model. The *z*-score and probability of event were calculated for each combination among the significant variables in predicting the outcome. The probability of the outcome of interest, the *Z* value, was then transformed into the probability of event using the following link function: P[event] = e^z^/1 + e^z^. This probability value ranges from 0 to 1.

The model was evaluated using sensitivity and specificity analysis based on the optimal cutoff derived from the probability of event and also based on probability of event with 0.5.

All analyses were carried out using SPSS (IBM Corporation, released 2011, IBM SPSS Statistics for Windows, Version 20.0. Armonk, NY: IBM Corp.) and Microsoft Office Excel 2007.

## 3. Results

There were 70,889 registered T2DM patients in the registry. After excluding the duplicate cases, 69,555 records were analyzed. Out of 69,555 records, 14.9% T2DM patients died within five years. The mortality rate was 1.4 persons per 100 person-years. The main causes for the mortality were diseases of the circulatory system (28.4%), infectious and parasitic diseases (19.7%), and respiratory system (16.0%). Deaths due to endocrine, nutritional, and metabolic diseases were ranked at fourth (9.6%) (Table 1). Based on univariate analysis, eight factors have sizeable odds ratio (more than 2.0) appeared in any of the category of the independent variables in predicting mortality within five years (Table 2).

All the effect sizes based on model 1 and model 2 were almost similar except that odds ratios for age group based on earlier data (60.0% of consecutive subjects from the first record) were almost double compared with the later data (40.0% of remaining consecutive subjects). However, the linear association can be observed where the older the subjects, the higher likelihood to die within five years. In general, the result based on later data was comparable with the earlier data. The almost similar coefficients that were derived from model 1 and model 2 have indicated that the internal validity is assumed. The model 3 was analyzed which consisted of all records, and the coefficient from this model was used to develop the screening tool to screen high-risk patients (Table 3). The model 3 reported the Nagelkerke R-square with 0.113, and the coefficient of receiver operating characteristic (ROC) of the probability of event towards the outcome was 71.0% (70.1%, 71.9%).

The coefficients and the revised coefficients for model 3 with examples of how the model was applied was presented in Table 4. The revised coefficients were proposed to simplify the exact coefficient with only one decimal point. The revised coefficients will be easier to be remembered and at the same time performed as good as if the original coefficients were used. Elderly (>65 years) and underweight patients with duration of diabetes more than 10 years have moderate probability to die (probability of event > 0.50) within five years if this group has at least one diabetes complication.

Sensitivity and specificity analysis were presented in Table 5. The optimal cutoff was 0.096 (or 0.10) with sensitivity and specificity of 70.2% and 61.3%, respectively. The probability of more than 0.5 was recommended as cutoff to screen high-risk patients with a specificity of 99.8% and negative predicted value of 88.4%. Patients with probability of event more than 0.5 are expected having odds ratio of at least six times more likely to predict death within five years.

## 4. Discussions

According to recent classification by WHO, “cardiovascular disease refers to several types of conditions affecting the heart and blood vessels, also known as the circulatory system. Some common cardiovascular diseases and conditions include heart disease, stroke and hypertension, also known as high blood pressure” [16]. Cardiovascular diseases (CVDs) continue to be the number one killer for diabetes patients [17–19], followed by infections.

It is known that patients with diabetes are at risk of developing wounds and sores that are difficult to heal well, and hence, infections have high tendency to become severe and progress faster; it is also difficult to treat. In severe cases, patients develop sepsis which can lead to septic shock. Previous studies have reported that among diabetes population, sepsis has become one of the leading causes of death in intensive care units (ICUs) [20]. CVDs and infections can be considered as unrelated causes of deaths for diabetes patients. The cause of death directly due to diabetes was ranked at fourth. This has showed that the risk of mortality among diabetes patients is highly driven by the development of chronic complications [21–23] due to diabetes.

However, the ranking for cause of death among T2DM varies between countries especially between developing countries and developed countries. For example, in Japan, the leading cause of death among patients with T2DM was malignant neoplasia and then followed by vascular diseases and infectious [24]. However, in the present study, the main cause of death among patients with T2DM in Malaysia is disease of circulatory system. This could be explained by the difference in access to treatment in developing countries. Cardiovascular disease is preventable and is treatable with access to immediate medical care upon cardiovascular events; therefore, with adequate medical care, premature deaths from cardiovascular disease can be prevented. In developed countries, majority of subjects have better access to treatment and the pattern is the same in the US [7].

Another finding is this study was that obese patients have a lower risk of dying within 5 years compared with patients with normal BMI. This can be explained by the distribution of age group and BMI status in our data based on this cohort; majority of patients with normal group were among the elderly (65 years and above). This finding can possibly be explained by the obesity paradox in which a higher BMI is associated with better outcome in several chronic diseases and health circumstances, for example, higher BMI value were independently associated with lower risk of death in heart failure patients in the US [25]. It was known that one of common diabetes complications on poorly controlled diabetes is weight loss which is associated with increased morbidity and mortality [26]. Therefore, in our T2DM population, patients with normal BMI reported to have higher risk of dying within 5 years compared with obese patients likely due to this reason.

This study has found that older age, longer duration of being diagnosed with diabetes, underweight, and diabetic complications as the risk factors towards mortality within five years. This finding is consistent with previous studies [8–11]. This study has some advantage in terms of simplicity of the model, which only requires basic demographic profile and clinical parameters. Blood investigations that were taken at baseline such as for blood glucose and lipids were less useful in predicting the outcome. Using eight variables which were all in categorical form, a logistic regression model was successfully developed. Stating the risk factors is useful; however, formulating a predictive model from the risk factors would ease the clinicians in prognostication of each patient [27–29].

Previous studies regarding modelling mortality for T2DM were developed based on cox regression models [8–11]. The cox regression model is very useful since the endpoint incorporates both time and event and it is usually very practical when the event is rare. On the other hand, this study proposed a logistic regression model where the duration was treated as part of the contributing factor, and the endpoint was mortality within five years. Without using complicated computer programming, the probability of event can be calculated based on the coefficients which are presented in excel as in Table 4. The logistic regression model has been used widely in clinical research to determine association between factors and outcomes [12–14]. If the effect sizes of the contributing factors are satisfactorily high, then it is worth to pursue the model for prediction [29].

Based on this logistic regression model, this study proposed a cutoff of probability of event with 0.5 and more to be used to screen high-risk patients. Some studies may sacrifice the specificity value to increase the sensitivity value of the model or vice versa [30, 31]. However, with probability of event of more than 0.5, the model can determine mortality within five years at least six times more likely compared with probability of event of lower than 0.5. Therefore, patients with probability of event 0.5 and above should receive closer attention. The majority of these patients (with probability of event 0.5 and above) are elderly, underweight, longer duration of being diagnosed with diabetes, and at least with one chronic diabetes complication such as ischaemic heart disease (IHD), cerebrovascular disease, nephropathy, and foot problems.

The strength of this study is that this is a large cohort data which analyzed the incidence of all cause of death based on the latest ICD-10 categorization. There are limited data on this topic in the Southeast Asian population. In Malaysia, this is the first time that the all cause of death was presented for T2DM patients and can serve as a baseline data for future comparison or cross-country comparisons. In addition, the factors associated towards mortality within 5 years among prevalent of T2DM were successfully determined, and a score model was developed which can be used as a screening tool to determine high-risk patients who require additional attention.

This study however has several limitations. The variables that were examined as the predictors were based on variables that were observed during the notification period. Some variables have missing values more than 50.0% such as status of retinopathy (data completeness with 49.4%). However, the analysis for multivariate analysis was still derived from a large dataset. Previous studies have shown that when very large sample sizes are used, the statistics are likely to represent the parameter in the intended population [32, 33]. Hence, the result of this study is likely to be able to infer to the larger population.

In addition, there were 3323 causes of death which were not verified, and this was probably due to their cause of deaths were not medically verified or family members requested for autopsy not to be conducted [34]. For future study, the authors recommended for the coefficients based on model 3 to be validated using external data to determine the robustness of the model to screen high-risk patients with prevalent T2DM.

In summary, this study found that the mortality within five years among T2DM patients were higher in older age group, longer duration of being diagnosed with diabetes, underweight, and diabetes complications. Concerning the model has low specificity, the present paper proposed the prognostic model to be used as a screening tool to classify prevalent T2DM patients who are at risk for mortality within five years.

## Figures and Tables

**Table 1:** Specific causes of death among prevalent T2DM patients within five years' period.

Number	Causes of death	*n*	%^a^
1	Diseases of the circulatory system	3003	28.4
2	Certain infectious and parasitic diseases	2077	19.7
3	Diseases of the respiratory system	1686	16
4	Endocrine, nutritional, and metabolic diseases	1010	9.6
5	Neoplasms	741	7.0
6	Diseases of the genitourinary system	613	5.8
7	Diseases of the digestive system	427	4.0
8	External causes of morbidity and mortality	276	2.6
9	Injury, poisoning, and certain other consequences of external causes	224	2.1
10	Diseases of the skin and subcutaneous tissue	172	1.6
11	Diseases of the nervous system	172	1.6
12	Diseases of the musculoskeletal system and connective tissue	83	0.8
13	Diseases of the blood and blood-forming organs and certain disorders involving the immune mechanism	62	0.6
14	Others	11	0.1
	Not elsewhere classified	3323	

^a^The percentage was calculated without including the cause of death with not elsewhere classified.

**Table 2:** Result of univariate association between factors and status of death within five years.

Factors	Completeness of data	Category	Died within 5 years				*p* value	Crude odds ratio >2.0
			No		Yes			
	%		*n*	%	*n*	%		
Age group (years)	100.0	<40	3154	5.3	100	1.0	<0.001	Yes
		40–64	41,837	70.7	4479	43.3		
		65 and above	14,226	24.0	5759	55.7		

Gender^a^	99.8	Male	23,095	39.1	5284	51.2	<0.001	No
		Female	36,027	60.9	5042	48.8		

Ethnicity^a^	99.8	Malay	36,997	62.6	6048	58.6	<0.001	No
		Chinese	10,836	18.3	2388	23.1		
		India	10,700	18.1	1813	17.6		
		Others	589	1.0	69	0.7		

BMI category (WHO)	77.1	Underweight	592	1.3	295	4.6	<0.001	Yes
		Normal	14,631	31.1	2842	43.9		
		Overweight	19,588	41.6	2207	34.1		
		Obese	12,309	26.1	1134	17.5		

Duration of disease (years)	82.1	<5	26,130	52.7	2898	38.6	<0.001	Yes
		5 to 10	17,028	34.3	2786	37.2		
		>10	6444	13.0	1815	24.2		

Hypertension	82.5	Yes	34,505	69.2	5892	78.1	<0.001	No

Dyslipidemia	82.5	Yes	23,248	46.6	3380	44.8	0.003	No

Status of HbA1c (%)	53.2	≤7.0	11,064	33.8	1380	32.0	0.015	No

Status of systolic (mmHg)	80.7	≤130	22,514	45.9	2763	39.0	<0.001	No

Status of diastolic (mmHg)	80.7	≤80	31,148	63.5	4744	66.9	<0.001	No

Status of LDL (mmol/L)	55.2	≤2.6	10,587	31.1	1372	31.2	0.960	No

Status of HDL (mmol/L)	53.4	≥1.1	23,364	71.1	2966	69.2	0.009	No

Status of Tg (mmol/L)	65.3	≤1.7	22,399	55.8	2836	53.7	0.005	No

Retinopathy	49.4	Yes	2456	8.1	621	14.9	<0.001	Yes

Ishaemic heart disease	58.7	Yes	1673	4.7	600	11.7	<0.001	Yes

Cerebrovascular	62.8	Yes	411	1.1	206	3.8	<0.001	Yes

Nephropathy	59.3	Yes	4068	11.3	1063	20.6	<0.001	Yes

Foot problem	64.7	Yes	1897	4.8	576	10.2	<0.001	Yes

^a^Gender and ethnicity cannot be imputed because there are no identifiers (name and identification card number) during the data export.

**Table 3:** Association of selected factors toward status of death within five years.

Factors	Category	Model 1^a^				Model 2^b^				Model 3^c^: all subjects			
		*p* value	OR^d^	95% CI		*p* value	OR^d^	95% CI		*p* value	OR^d^	95% CI	
Age group	<40	ref. gp				ref. gp				ref. gp			
	40 to <65	<0.001	5.5	2.8	10.7	<0.001	2.8	1.8	4.6	<0.001	3.7	2.5	5.5
	65 and more	<0.001	16.1	8.3	31.3	<0.001	8.8	5.5	14.2	<0.001	11.2	7.6	16.5

BMI category	Underweight	<0.001	3.8	2.9	5.1	<0.001	3.5	2.6	4.8	<0.001	3.7	3.0	4.6
	Normal	<0.001	1.4	1.2	1.7	<0.001	1.5	1.3	1.7	<0.001	1.5	1.3	1.6
	Overweight	0.708	1.0	0.8	1.1	0.882	1.0	0.8	1.2	0.733	1.0	0.9	1.1
	Obese	ref. gp				ref. gp				ref. gp			

Duration of disease	<5	ref. gp				ref. gp				ref. gp			
	5–10	0.030	1.1	1.0	1.3	0.001	1.2	1.1	1.4	<0.001	1.2	1.1	1.3
	>10	<0.001	1.4	1.2	1.6	0.002	1.3	1.1	1.6	<0.001	1.3	1.2	1.5

Retinopathy	Yes	0.005	1.3	1.1	1.5	0.001	1.4	1.2	1.8	0.001	1.3	1.2	1.5
	No	ref. gp				ref. gp				ref. gp			

IHD	Yes	0.006	1.4	1.1	1.7	<0.001	1.8	1.4	2.3	<0.001	1.5	1.3	1.8
	No	ref. gp				ref. gp				ref. gp			

Cerebrovascular	Yes	<0.001	2.4	1.6	3.5	0.007	2.0	1.2	3.2	0.007	2.2	1.6	3.0
	No	ref. gp				ref. gp				ref. gp			

Nephropathy	Yes	<0.001	1.7	1.4	1.9	0.001	1.4	1.1	1.7	0.001	1.5	1.4	1.7
	No	ref. gp				ref. gp				ref. gp			

Foot problem	Yes	<0.001	1.8	1.4	2.2	0.001	1.5	1.2	1.9	0.001	1.7	1.4	2.0
	No	ref. gp				ref. gp				ref. gp			

^a^Model 1: analyzed based on 60% consecutive data from the first subject (eligible data for multivariate was derived from 13,535 out of 27,822 records). ^b^Model 2: analyzed based on remaining 40% of the consecutive subjects (eligible data for multivariate was derived from 15,310 out of 15,310 records). ^c^Model 3: analyzed based on all dataset (eligible data for multivariate was derived from 28,845 out of 69,555 records). ^d^OR referred as odds ratio.

**Table 4:** The examples in calculating the probability of event based on the coefficients in the model 3.

Factors	Category	Coefficient	Coeff. Rev.^a^	Patient 1		Patient 2		Patient 3		Patient 4		Patient 5	
				M1^b^	M2^c^	M1^b^	M2^c^	M1^b^	M2^c^	M1^b^	M2^c^	M1^b^	M2^c^
Age group	<40	ref gp^d^	ref gp^d^										
	40 to <65	1.317	1.300							x	x	x	x
	≥ 65	2.415	2.400	x	x	x	x	x	x				

Duration of disease	<5	ref gp^d^	ref gp^d^										
	5–10	0.155	0.200							x	x		
	>10	0.288	0.300	x	x	x	x	x	x			x	x

BMI	Underweight	1.307	1.300	x	x	x	x	x	x	x	x	x	x
	Normal	0.377	0.400										
	Overweight	−0.019	−0.020										
	Obese	ref gp^d^	ref gp^d^										

Retinopathy	Yes	0.274	0.300			x	x			x	x		

Ishaemic heart disease	Yes	0.424	0.400					x	x			x	x

Cerebrovascular	Yes	0.783	0.800									x	x

Nephropathy	Yes	0.433	0.400									x	x

Foot problem	Yes	0.504	0.500										

Constant		−4.084	−4.100										

*Calculate probability of event*													
Step 1. Calculate *z*-score				−0.073	−0.100	0.201	0.200	0.351	0.3	−1.030	−1.000	0.469	0.400
Step 2. Calculate exponential of *z*				0.929	0.905	1.222	1.221	1.420	1.350	0.357	0.368	1.598	1.492
Step 3. Calculate 1 + exponential of *z*				1.929	1.905	2.222	2.221	2.420	2.350	1.357	1.368	2.598	2.492
Step 4. Calculate probability of event				0.482	0.475	0.550	0.550	0.587	0.574	0.263	0.269	0.615	0.599

^a^Coeff. Rev. refers to revised coefficient. ^b^M1 refers to model 1 where the probability of event was calculated based on exact coefficients. ^c^M2 refers to model 2 where the probability of event was calculated based on revised coefficients. ^d^ref gp refers to reference group.

**Table 5:** Sensitivity and specificity analysis and logistic regression of probability of event of ≥0.5 in predicting mortality within five years.

Probability of event	Description	Died within 5 years		Sensitivity (95% CI)	Specificity (95% CI)	Logistic regression
		No	Yes			OR (95% CI), *p* value
<0.5	Predict to survive within 5 years	25,398 (99.8%)	3349 (98.7%)	1.33% (0.97%, 1.77%)	99.79% (99.73%, 99.84%)	6.4 (4.3, 9.6), *p* < 0.001
	
≥0.5	Predict to die within 5 years	53 (0.2%)	45 (1.3%)			
	

Note: positive predicted value (PPV) was 45.9% (36.4%, 55.8%). Negative predicted value (NPV) was 88.4% (88.3%, 88.4%).

## Data Availability

The authors could not release the data because the data is kept in a national registry and maintained by the Ministry of Health. However, data request can be made through the local authority: Non-Communicable Disease (NCD) Section, Disease Control Division, Ministry of Health Putrajaya, Level 2, Block E2, Complex E, Federal Government Administration Centre, 62,590 Putrajaya, Malaysia.
